# Mechanistic Aspects of Inflammation and Oxidative Stress and Their Association With Thyroid Cancer Risk

**DOI:** 10.1002/cam4.71030

**Published:** 2025-07-07

**Authors:** Bo‐Tao Zhang, Mao Guo, Liu‐Rui Yang, Yang Zeng, Jun Jiang

**Affiliations:** ^1^ Department of Pain Management Luzhou People's Hospital Luzhou China; ^2^ Department of General Surgery (Thyroid Surgery) The Affiliated Hospital of Southwest Medical University Luzhou China; ^3^ Medical Insurance and Price Management Department Luzhou People's Hospital Luzhou China; ^4^ Department of Orthodontics The Affiliated Stomatology Hospital of Southwest Medical University Luzhou China

**Keywords:** adipocytokines, chronic inflammation, obesity, oxidative stress, reactive oxygen species, thyroid cancer

## Abstract

**Aim:**

To investigate the mechanistic aspects of inflammation and oxidative stress and their association with thyroid cancer risk.

**Methods:**

We have systematically searched the PubMed and Web of Science databases to perform a comprehensive analysis of the pathogenic mechanisms that link obesity, inflammation, and oxidative stress to thyroid cancer.

**Results:**

Chronic inflammation, a well‐known risk factor for cancer progression, is a hallmark characteristic of obesity. Multiple mechanisms may mediate the association between thyroid cancer and obesity. As a crucial endocrine organ, adipose tissue regulates tumor behavior, inflammation, and the tumor microenvironment through adipokines, including leptin, adiponectin, as well as chemokines. Excessive fat accumulation leads to an increase in pro‐inflammatory factors, which in turn result in systemic inflammation that can further influence tumor growth and development. Oxidative stress, characterized by an imbalance between the production of reactive oxygen species (ROS) and their elimination by antioxidants, can potentially result in cellular damage and destruction. With the accumulation of ROS, it participates in numerous diseases, such as cancer and inflammation. The inflammatory condition of the thyroid gland serves as a classical source of ROS, which may, in turn, promote the development of thyroid tumors.

**Conclusion:**

While various mechanisms may elucidate the relationship between inflammation, oxidative stress, and the risk of thyroid cancer, the interplay among these factors is intricate and cannot be solely attributed to a singular pathway. This review provides further insight into a strategy for the prevention and treatment of thyroid cancer, highlighting the significance of combinatorial approaches that target multiple pathways in antitumor therapy.

## 1 Introduction

The global surge in thyroid cancer incidence presents an urgent public health challenge, with cases rising by 3.6% annually since 2015—a rate surpassing most solid tumors (WHO GLOBOCAN 2020). Particularly alarming is its disproportionate impact on women under 49, who account for 80% of new diagnoses in developed nations (SEER Program, NCI) [1]. If the current upward trend continues, thyroid cancer will be the fourth most common cancer globally by 2030 [2]. In the past few decades, technological advancements in imaging diagnosis have significantly facilitated the early detection of thyroid cancer and nonpalpable thyroid nodules [3]. Nevertheless, there has been a notable rise in the incidence of tumors of varying sizes [4], indicating that early detection and potential over‐diagnosis alone cannot sufficiently account for the high prevalence of thyroid cancer [5]. These findings suggest the possibility that additional factors, such as lifestyle and environment, may also play a role in the development of thyroid cancer. Despite extensive research efforts, the etiology of thyroid cancer remains unclear. Currently, several risk factors such as obesity, family history, and ionizing radiation exposure in childhood have been identified as contributing to an increased risk of developing thyroid cancer [6].

Obesity is a chronic condition characterized by the accumulation of adipose tissue. This tissue serves not only as energy storage but also functions as an endocrine organ that secretes various adipokines, including leptin, resistin, and adiponectin [7]. Dysregulation of adipose tissue is often accompanied by an imbalance in multiple adipocytokines, which can contribute to further metabolic disorders and chronic inflammation [8, 9]. These alterations are associated with the initiation, progression, and metastasis of tumors [10]. Existing epidemiological studies have shown that obesity‐related comorbidities, including altered adipokine secretion, chronic low‐grade inflammation, and abnormal insulin resistance, may represent potential mechanisms underlying the association between obesity and thyroid cancer [11, 12]. The relationship between obesity and thyroid cancer has aroused interest in investigating the role of adipokines in these malignant tumors.

Oxidative stress is a physiological state of imbalance between the intracellular oxides and anti‐oxidant systems, which can result in the production of various reactive oxygen species (ROS) in response to both internal and external stressors [13]. The generation of ROS may be facilitated by numerous enzyme complexes, including NADPH oxidases (NOX), nitric oxide synthases, cytochrome P‐450 enzymes, and the mitochondrial electron transport chain [14]. The regulation of ROS levels is essential for maintaining cellular homeostasis, as varying concentrations of ROS are implicated in distinct biological processes [15]. Extensive experimental evidence has established a significant correlation between oxidative stress and the development of cancer. In humans, the thyroid gland is the largest endocrine organ, responsible for the secretion of the thyroid hormones (TH). The synthesis of TH is a complex, multistep process regulated by the hypothalamus–pituitary–thyroid axis via thyroid‐stimulating hormone (TSH) [16]. TSH can stimulate the production of hydrogen peroxide (H_2_O_2_) in the thyroid gland, which acts as a stable, mild oxidant and is essential for the iodination of thyroglobulin, ultimately leading to the production of TH [17]. Under physiological conditions, ROS plays a crucial role in TH synthesis. However, elevated levels of oxidative stress can induce cellular inflammation within the thyroid gland [18]. Consequently, the thyroid gland is particularly susceptible to oxidative stress damage due to the oxidative nature of TH synthesis. Recent studies have indicated a correlation between oxidative reactions and the occurrence and progression of thyroid cancer [19, 20]. It has been reported that a significant elevation in serum oxidant levels in cases of thyroid cancer may serve as a distinguishing marker for differentiating between benign and malignant thyroid tumors [21].

Although the epidemiological association between obesity and thyroid cancer risk has been widely documented, the molecular mechanisms connecting metabolic disorders and carcinogenic processes remain a black box. This review synthesizes current evidence elucidating the pathogenic mechanisms by which obesity‐induced chronic inflammation and oxidative stress dysregulation promote thyroid carcinogenesis, focusing on the pivotal role of adipokine‐mediated microenvironmental reprogramming in the malignant transformation of thyroid cells, and emerging clinical evidence supporting the diagnostic/prognostic utility of adipokine signaling as molecular biomarkers in thyroid oncology in order to provide a novel strategy for research and clinical practice in the treatment of thyroid cancer.

## 2 Adipocytokines and Thyroid Cancer

**FIGURE 1:**
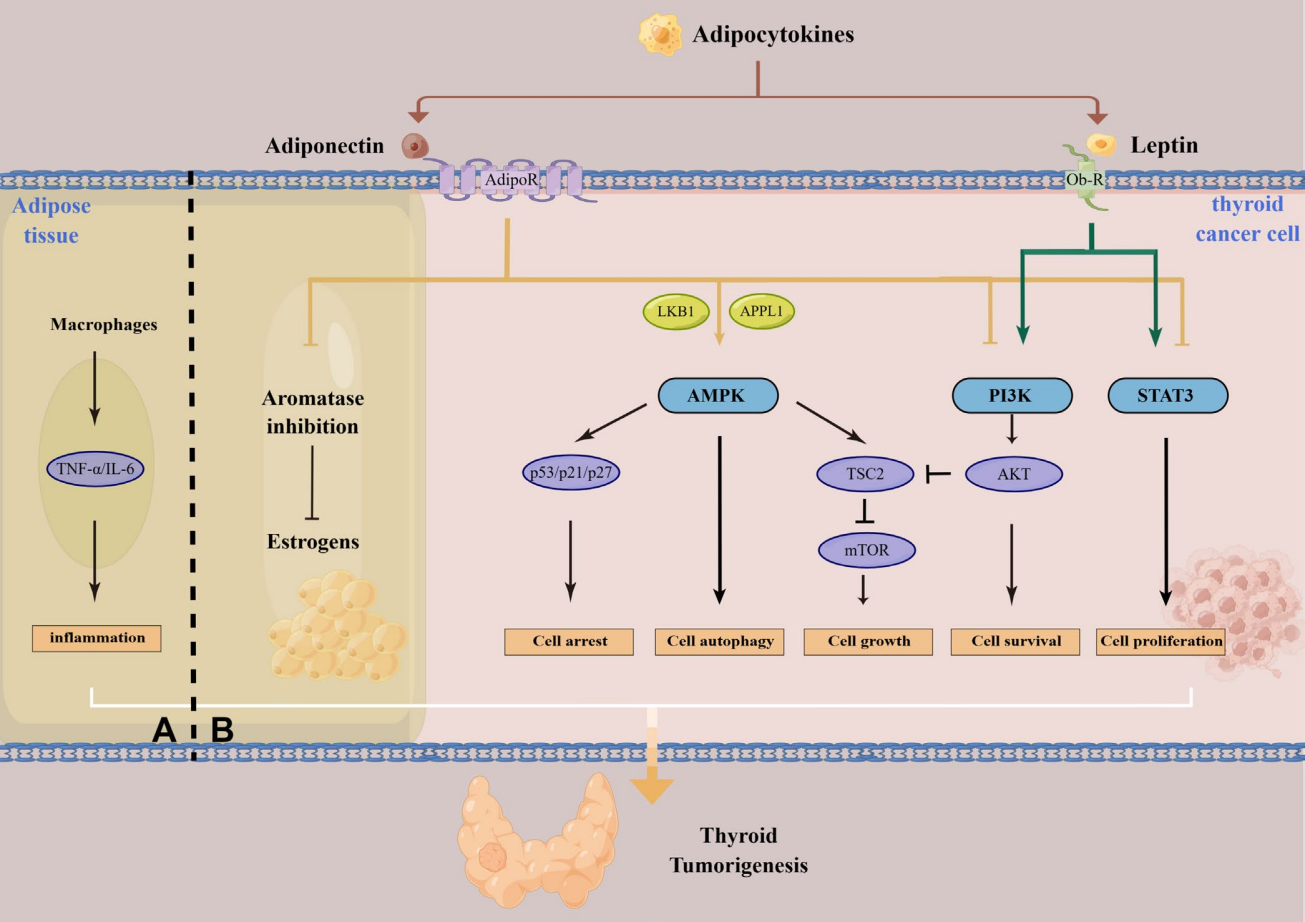
The influence of adipose tissue on thyroid cancer cells. (A) The pro‐inflammatory cytokines secreted by macrophages are involved in the pathogenesis of thyroid cancer. (B) Adipokines produced by adipose tissue interact with their receptors to modulate signaling pathways in thyroid cancer.

Recent studies have demonstrated that adipocytokines play a pivotal role in thyroid carcinogenesis. These adipocytokines can influence thyroid cancer cells through autocrine, paracrine, and endocrine pathways, thereby facilitating the progression of thyroid cancer [22]. Several hypotheses have been proposed to explain the underlying mechanisms of this association (Figure 1), including inflammation, oxidative stress, and the dysregulation of adipokines (mainly adiponectin and leptin) [23].

### 2.1 Adiponectin (APN)

Adiponectin, the most abundant adipokine in plasma synthesized by adipose tissue, has been proposed to possess anti‐inflammatory, anti‐apoptotic, and insulin‐sensitizing properties [24, 25]. Epidemiological evidence has demonstrated that APN levels in thyroid cancer patients are significantly lower than those in healthy individuals, and there exists a negative correlation between APN levels and obesity [26, 27]. Although APN has been shown to inhibit thyroid cancer cell growth and metastasis, the underlying molecular mechanism remains unclear. Previous studies have suggested that APN exerts its anti‐neoplastic effects on thyroid cancer through two main mechanisms, which will be discussed below.

#### 2.1.1 Direct Pathways

The primary direct mechanism through which APN exerts a protective effect on thyroid cancer cells is the activation of AMP‐activated protein kinase (AMPK) via adiponectin receptors 1 and 2 [28]. APN enhances AMPK activity by elevating AMP levels and engaging various cellular mediators, including liver kinase B1 (LKB1), calcium‐dependent proteins, and adaptor protein APPL‐1 [29, 30]. LKB1 is an important upstream activator of AMPK, and the activation of AMPK has been shown to negatively affect cancer progression by modulating several key mechanisms that regulate cellular development and growth [31, 32]. On the one hand, LKB1‐dependent AMPK activation can directly result in the phosphorylation of the tumor suppressor TSC2 (tuberous sclerosis complex 2), which inhibits mTOR activation and exerts an antitumor effect [33]. On the other hand, AMPK activation can promote the expression of crucial molecules involved in cell differentiation and apoptosis [34, 35]. In fact, APN treatment has been demonstrated to stimulate AMPK in the thyroid [36], breast [37], and endometrial cancers [38], thereby mediating tumor cell growth inhibition.

Likewise, the phosphoinositide 3‐kinase/protein kinase B (PI3K/AKT) pathway is also involved in the regulation of cellular physiology. It has been identified that APN initiates a sequence of events that lead to apoptosis and/or tumor growth inhibition through the suppression of the PI3K/AKT pathway. Interestingly, AKT has the capacity to phosphorylate and inhibit TSC2, which in turn diminishes the function of AMPK [29]. Moreover, APN can modulate the STAT3 pathway, which is activated via adipokine‐induced Janus kinase (JAK) phosphorylation, thereby influencing various cellular processes associated with cancer initiation and development [39, 40].

#### 2.1.2 Indirect Pathways

APN can exert its anti‐neoplastic effects indirectly through different mechanisms, including insulin‐sensitizing and angiogenesis [41]. Research indicates that circulating adiponectin levels are inversely correlated with fasting plasma insulin while exhibiting a positive correlation with insulin sensitivity. It is well documented that insulin facilitates tumor cell proliferation and survival [42, 43]. Furthermore, it has been suggested that APN is a potent inhibitor of the PI3K/AKT pathway, which may attenuate the growth of tumor cells stimulated by insulin and cytokines [44].

The presence of blood vessels within tumors facilitates the delivery of nutrients and oxygen to cancer cells, thereby establishing angiogenesis as one of the hallmarks of cancer [45]. Therefore, inhibition of angiogenesis is regarded as a promising therapeutic strategy for suppressing tumor growth. Several studies suggest that APN may function as an effective angiogenesis inhibitor, capable of impeding tumor vascularization and suppressing tumor growth by modulating specific signaling pathways, such as the MAPK and cAMP/PKA pathways [46].

Despite the completion of numerous studies, research regarding APN and its signaling pathways in the context of thyroid cancer has advanced only minimally to date. Therefore, further investigations into APN and its pathways concerning their effects on cell function are warranted.

### 2.2 Leptin

Leptin, a product of the obesity gene, plays a key role in energy homeostasis [47] and is known to exert biological functions through the leptin receptor (Ob‐R) [48]. Both leptin and the Ob‐R have been detected in various malignant tumors, including those of the thyroid, breast, and colorectal regions. Given that elevated serum leptin levels are observed in obese patients, this adipokine may be associated with the onset and progression of thyroid cancer [23]. Fan et al. reported a strong correlation between leptin expression and Ob‐R expression in thyroid cancer [49]. Furthermore, several studies have indicated that increased expression of leptin and Ob‐R in papillary thyroid cancer (PTC) is directly linked to the aggressiveness of the neoplasm [49, 50].

It has been demonstrated that leptin exerts its effects via the PI3K/AKT and AKT/STAT3 pathways in PTC. An in vitro study revealed that leptin activates the PI3K pathway and promotes the phosphorylation of AKT, thereby triggering critical pathways linked to the proliferation of thyroid cancer cells [51]. Park et al. highlighted the effect of the STAT3 inhibitor S3I‐201 on the progression of thyroid cancer induced by a high‐fat diet (HFD) [52].

In conclusion, numerous studies have elucidated the role of leptin in thyroid cancer, demonstrating its capacity to enhance cell growth and survival through mechanisms that include the promotion of cell proliferation, the reduction of apoptosis, and the regulation of metabolic activity.

### 2.3 Resistin

In addition to alterations in adiponectin and leptin levels, elevated levels of other adipokines, such as resistin, interleukin‐6 (IL‐6), and tumor necrosis factor‐alpha (TNF‐α), are associated with an increased risk of cancer [53]. Resistin is a novel cytokine characterized by its pro‐inflammatory properties and is present in both murine and human systems. In rodent models, resistin is released by adipocytes, whereas in humans, it is secreted by mononuclear cells [54]. Although the precise molecular mechanisms of resistin remain incompletely understood, existing studies suggest a pro‐inflammatory role for this cytokine in humans. Resistin facilitates immune cell recruitment by stimulating pro‐inflammatory factor expression, which contributes to chronic subclinical inflammation often observed in metabolic disorders. Given its capacity to modulate the production of immune molecules and its indirect regulatory effects on the MAPK pathway, resistin has been the subject of investigation in various human cancers. Notably, resistin expression has been linked to breast, lung, hepatocellular, pancreatic, colorectal, and prostate cancers [55]. However, more research is required to understand the detailed mechanisms underlying thyroid cancer.

### 2.4 Estrogen

The association between thyroid cancer and gender has been well‐documented, with existing literature indicating that women exhibit a higher incidence of thyroid cancer [56, 57]. In obese women, there is an increased expression of aromatase in adipose tissue, which leads to a greater conversion of androgens to estrogens. Furthermore, aromatase activity is stimulated by various pro‐inflammatory factors, including IL‐1β, IL‐6, and TNF‐α, all of which are known to elevate with weight gain [58].

Banu et al. conducted a study that demonstrated the influence of both estradiol and testosterone on the proliferation of PTC and follicular thyroid cancer cell lines. This study found that the cell cultures tested positive for androgen and estrogen receptors (ERα and ERβ), which were up‐regulated by their respective ligands [59]. Multiple studies have shown the expression of ERs in thyroid cancer cells, indicating that these cells are more susceptible to the effects of ERs or estrogen [60, 61]. Specifically, estrogen has been related to the growth and progression of thyroid tumor cells, as well as alterations in various metabolic and angiogenic markers [62, 63, 64]. This evidence underscores the significant role of estrogen and ERs in thyroid cancer cells.

## 3 Inflammation and Thyroid Cancer

**FIGURE 2:**
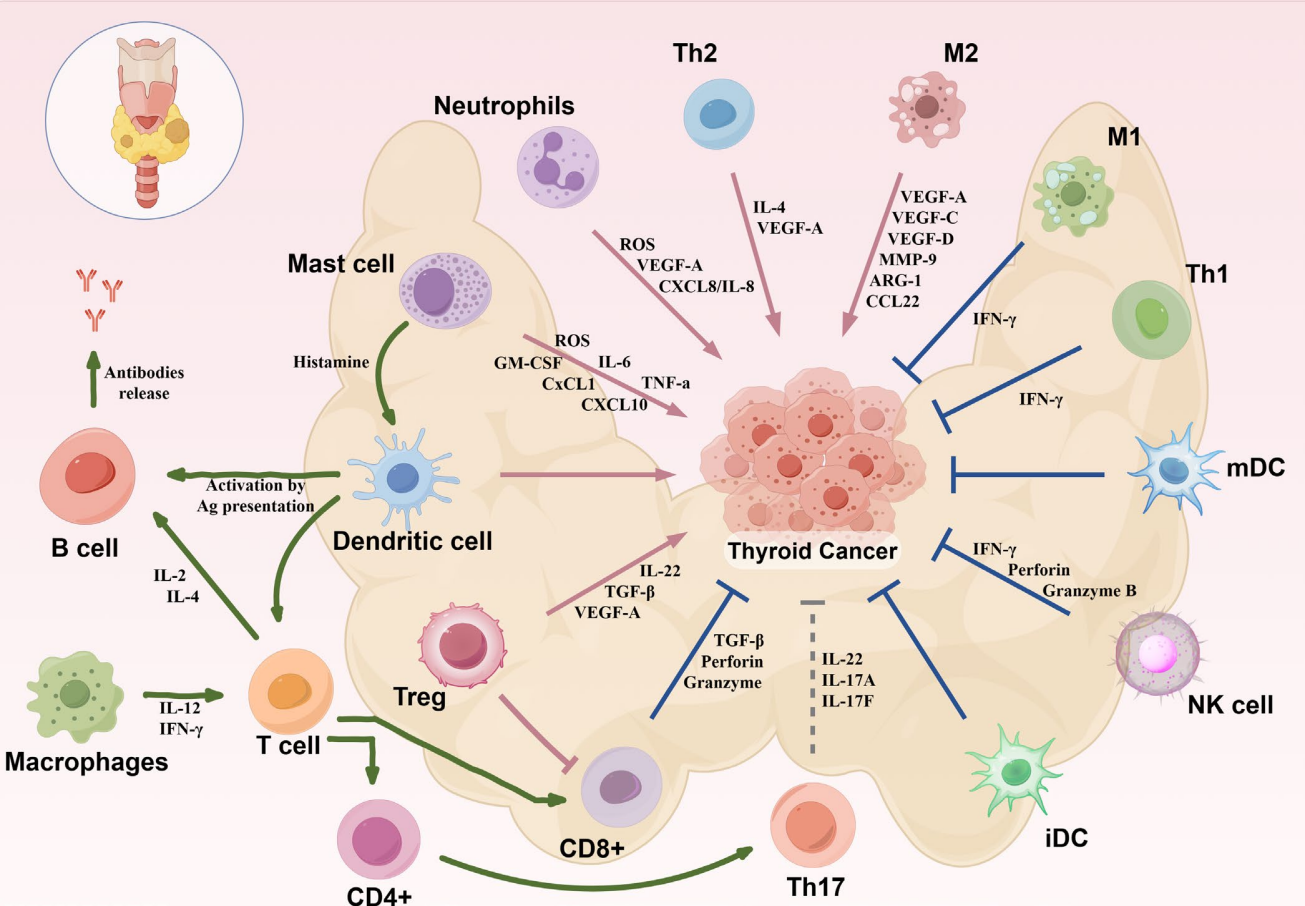
This schematic illustrates the interactions among tumor‐infiltrating immune cells and their interactions with thyroid cancer cells. T regulatory cells (Treg), T helper 2 (Th2) cells, tumor‐associated mast cells, tumor‐associated neutrophils, tumor‐associated macrophages, M2 macrophages, immature DCs (iDCs), along with their mediators, are implicated in promoting tumorigenesis in thyroid cancer. M1 macrophages, cytotoxic CD8 T cells, Th1 cells, natural killer (NK) cells, mature DCs (mDCs), and their mediators are associated with antitumorigenic effects. The roles of Th17 and Tc17 cells in tumor promotion or suppression are context‐dependent.

Obesity is associated with chronic low‐grade inflammation due to the production of inflammatory cytokines and the influx of immune cells, such as macrophages [65]. Recent research has increasingly presented compelling evidence suggesting that obesity‐associated inflammatory changes might promote the development of various cancers, including thyroid cancer (Figure 2), through multiple mechanisms [66].

### 3.1 Macrophages

Besides adipocytes, adipose tissue also contains abundant adipose‐tissue macrophages, which are a complex ensemble of various macrophage subtypes and are prevalent in the tumor‐adipose microenvironment to facilitate tumor progression [67]. Among the immune cell subsets in cancer, macrophages have garnered the most attention to date. There are two major types of macrophages: classically activated M1 macrophages and alternatively activated M2 macrophages [68]. M1 macrophages are generally regarded as pro‐inflammatory due to their capacity to release a substantial amount of pro‐inflammatory cytokines, including TNF‐α and IL‐6 [69].

TNF‐α is a systemic inflammatory cytokine that plays a role in the cytotoxic activity of other cytokines against tumor cells. Elevated circulating levels of TNF‐α in both obese rodents and humans suggest a potential relationship between obesity and tumorigenesis. TNF‐α exhibits anti‐proliferative activity in human PTC through a receptor‐mediated mechanism. However, exposure of PTC cells to TNF‐α has been associated with a progressive loss of this anti‐proliferative effect [70]. This phenomenon may represent a mechanism by which obesity‐induced high TNF‐α exposure contributes to the progression of thyroid tumors. While an appropriate concentration of TNF‐α may confer protective effects, excessive levels can be detrimental, potentially leading to resistance of cancer cells to TNF‐related apoptosis‐inducing ligand when apoptotic processes are impaired. Additionally, TNF‐α can stimulate the production of various vasoactive mediators, such as prostaglandin and interleukin [71], which may promote tumor proliferation and diminish immune function. TNF‐α can also induce elevated levels of vascular endothelial growth factor [72], which is essential for providing oxygen and nutrients to tumor cells and plays a critical role in tumor metastasis. Furthermore, results from clinical research studies provide evidence for basic research. Some prior studies have demonstrated that the serum TNF‐α levels in patients with PTC are significantly higher than those in patients with benign conditions, who in turn exhibit higher levels than healthy individuals [73, 74].

IL‐6, a pleiotropic cytokine, plays a crucial role in the initiation and progression of various types of cancers [75]. A strong positive correlation has been established between serum IL‐6 levels and poor prognosis in patients with specific tumor types [76, 77]. Current research indicates that serum IL‐6 levels are significantly elevated in individuals with malignant thyroid tumors compared to healthy controls [78]. For in vitro studies, IL‐6 was also found to be expressed in thyroid cancer [79]. The underlying mechanism can be elucidated through the work of Couto et al., who identified that the IL‐6/gp130/JAK pathway is responsible for the activation of the STAT3 pathway in a human thyroid cancer‐derived cell line [80]. Notably, STAT3 deficiency resulted in larger and more proliferative tumors, suggesting that STAT3 may function as a negative regulator of tumor growth, at least in the context of thyroid cancer. Moreover, IL‐6 has been associated with malignancy in thyroid nodules and tumor invasiveness [81]. These findings strongly support the significant role of IL‐6 in thyroid cancer; however, the detailed mechanism by which IL‐6 exerts its effects remains to be elucidated.

### 3.2 T Cells

There is currently significant interest in the role of tumor T cells, especially given their critical function as mediators of immunotherapies. Reports indicate that levels of exhausted and dysfunctional T cells are elevated in obese cancer patients [82, 83], suggesting that cancer immunotherapy may represent a promising therapeutic option for this population. In fact, both animal and human models have demonstrated that obesity is associated with an improved response to immune checkpoint therapies, including anti‐PD1 and anti‐CD8 treatments [82, 83, 84].

## 4 Oxidative Stress and Thyroid Cancers

Oxidative stress represents an imbalance between ROS production and their neutralization by antioxidants, which can result in cellular damage and destruction. This phenomenon is associated with a variety of diseases, including aging, cancer, and inflammation [85]. In tissues, elevated intracellular levels of ROS, which may arise from tumorigenic events such as metabolic alterations or oncogene activation, can facilitate tumor initiation or progression [86].

Most previous studies have examined the association between oxidative or anti‐oxidant molecules in various individuals and the incidence of thyroid cancer, thereby providing further evidence to suggest a relationship between oxidative stress and this malignancy [87, 88, 89]. Wang et al. conducted a more in‐depth analysis of this issue by assessing the total oxidant status and oxidative stress index in patients, contributing additional evidence regarding the significance of oxidative stress in relation to thyroid cancer [90]. Although a direct causal relationship between oxidative stress and thyroid cancer has yet to be thoroughly investigated, existing research appears to have firmly established a correlation between the two.

### 4.1 Oxidative DNA Damage

During oxidative stress, elevated ROS levels induce oxidative damage to macromolecules such as lipids, proteins, and DNA, which serve as an initial step in tumorigenesis [91]. ROS can interact with base and sugar components of DNA, resulting in various types of lesions such as oxidized bases, adducted bases, DNA strand breaks, and modifications to the sugar moiety, which are addressed by distinct DNA damage repair pathways [92]. If left unrepaired, such lesions can alter genetic information, ultimately leading to the loss of genome integrity.

Among the DNA bases, guanine exhibits the highest susceptibility to oxidation [93]. Although various types of oxidative DNA damage products have been identified, 8‐hydroxy‐2′‐deoxyguanosine (8‐OHdG) has been the focus of extensive research and is recognized as the most representative biomarker of DNA damage [94, 95]. A study showed that 8‐OHdG levels may serve as a potential carcinogenic marker for multinodular goiters [96]. In PTC patients, 8‐OHdG levels in the preoperative PTC group were significantly elevated compared to those in the postoperative PTC group and the healthy control group. In addition, serum 8‐OHdG levels in PTC patients before surgery exhibited a positive correlation with the oxidative stress index and lipid hydroperoxide levels, while demonstrating a negative correlation with total antioxidant status levels. These findings suggest the presence of significant oxidative DNA damage and compromised antioxidant status in patients with PTC [97].

### 4.2 Estrogen

The incidence of thyroid cancer has an obvious gender tendency, with a higher prevalence observed in women. Estrogen serves as a potent growth factor for both benign and malignant thyroid cells, facilitating processes, such as cancer cell migration, apoptosis, and angiogenesis [98]. It executes its growth‐promoting effect through a pathway involving a membrane‐bound estrogen receptor (ER) [99]. Recent evidence indicates that ERα can enhance cell proliferation and inhibit apoptosis in PTC cells by inducing autophagy via the activation of ROS and ERK1/2 pathways [100]. Thus, ROS plays a key role in triggering autophagy in PTC cells, thereby promoting the progression of PTC by augmenting autophagic processes [63].

### 4.3 Hypoxia

Hypoxia, a condition characterized by an imbalance between oxygen consumption and supply, is a stress that influences several physiological and pathological processes [101]. As a hallmark of the tumor microenvironment, hypoxia is commonly induced by the rapid proliferation of cancer cells, which outpace the available blood supply, thereby exhausting the oxygen and nutrients supplied to cells [102]. ROS are elevated in nearly all cancer types under hypoxic conditions and play a significant role in the promotion of tumor development and progression [103]. A study conducted by Liao et al. indicates that hypoxia‐induced migration and invasion of thyroid cancer cells are related to the production of ROS, and hypoxia can significantly enhance ROS production, thereby augmenting the migratory and invasive capabilities of thyroid cancer cells [104].

In summary, ROS plays a crucial role in cell migration and invasion, thereby facilitating tumor progression and metastasis. Therefore, these findings establish a mechanistic connection between oxidative stress and thyroid cancer, suggesting that elevated oxidative stress may serve as a mechanism linking a risk factor, such as ionizing radiation, to the increased risk of thyroid cancer.

## 5 Sources of ROS in Thyroid Cancer

ROS are implicated in various cellular signaling pathways and functions, including cell proliferation and apoptosis [105]. If the mechanism connecting oxidative stress to thyroid cancer involves a positive role of ROS in the promotion of thyroid cancer, it is necessary to have a consistent source that can provide ROS within the thyroid cancer context.

### 5.1 NADPH Oxidase

It is widely accepted that the mitochondrial electron transport chain serves as the main source of ROS generation under physiological states [106]. However, ROS can also be generated by nonmitochondrial enzymes, which are regulated by growth factors and cytokines, particularly in pathological states [107]. Although several enzymes can generate ROS, NADPH oxidase (NOX) is specialized to generate ROS as its principal function [107, 108]. Among the seven proteins in the NOX family (NOX1‐5 and DUOX1‐2), the expression of DUOX1, DUOX2, and NOX4 has been extensively investigated in the thyroid gland [109].

**FIGURE 3:**
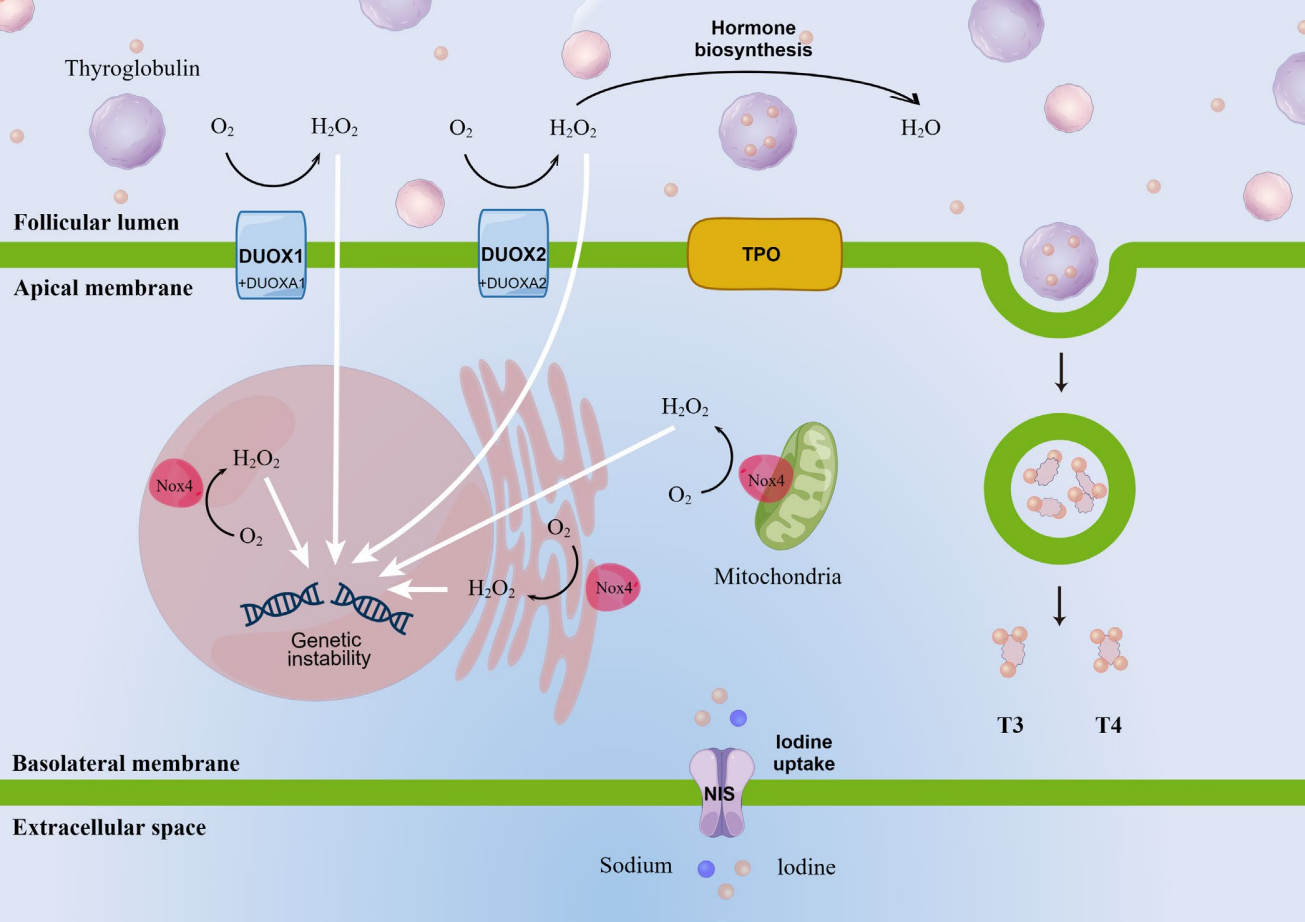
A hypothetical model elucidating the role of NADPH oxidase‐generated H_2_O_2_ in inducing DNA damage within thyroid cells is presented. During thyroid hormone biosynthesis, iodine is transported into thyroid follicular cells via the sodium‐iodine symporter (NIS), which is located on the basolateral membrane. The specific function of DUOX1 in thyroid biology remains to be fully elucidated; however, it is posited that DUOX1 may compensate for the deficiency of DUOX2. Under pathological conditions, H_2_O_2_ generated by DUOXs can diffuse across the apical membrane of thyroid cells, subsequently reaching the nucleus either directly or through the redox‐signaling pathway that may activate NOX4 in the nucleus and endoplasmic reticulum. The presence of NOX4 in the perinuclear region may elevate nuclear oxidative stress, thereby promoting oxidative damage to nuclear DNA and contributing to genetic instability.

DUOX was initially identified as a H_2_O_2_ generation system associated with thyroperoxidase (TPO) derived from thyroid epithelial cells, which is an important enzyme that catalyzes the biosynthesis of TH [110]. The interaction between DUOX and TPO is facilitated by H_2_O_2_, regulating extracellular H_2_O_2_ levels [111]. DUOX requires a specific maturation factor to exit the endoplasmic reticulum and subsequently translocate to the apical plasma membrane, where it forms a complex (Figure 3). This process is the basis of H_2_O_2_ production [112]. The complex at the plasma membrane serves a protective role for cells against oxidative stress‐induced damage. An analysis of The Cancer Genome Atlas (TCGA) data indicated that the expression levels of DUOX1 and DUOX2 were associated with tumor differentiation in the context of PTC; notably, lower expression levels were observed in tumors harboring BRAFV^600E^ mutations, which exhibited a lower degree of differentiation compared to other PTC tumors [113].

NOX4, initially identified in the kidney, exhibits higher expression levels compared to other NOX proteins. To date, the physiological role of NOX4 in the thyroid remains largely unexplored. Unlike DUOX, NOX4 is active not only in the plasma membrane but also within mitochondria and the nucleus [114]. Furthermore, in contrast to other DUOX enzymes, the expression of NOX4 is regulated by TSH and up‐regulated in thyroid cancer [115]. There is a correlation between elevated TSH levels and an increased risk of thyroid malignancy [116, 117]. As a ROS generation system regulated by TSH, NOX4 may be involved in this connection.

### 5.2 Inflammatory Environment

Thyroid cancer, similar to many other malignancies, is characterized as an inflammatory disease [118]. It is well‐known that there is a significant presence of inflammatory cells is observed in thyroid cancer [119], and certain inflammatory cytokines such as TGF‐β1 and thrombospondin‐1 play crucial roles in the process of thyroid tumorigenesis [120, 121]. Consequently, the inflammatory state of the thyroid gland, along with the subsequent evolution of the inflammatory tumor microenvironment, appears to be a fundamental aspect of the pathogenesis of thyroid cancer. The BRAF mutation, recognized as the most prevalent genetic alteration associated with thyroid tumor progression and aggressiveness [122], may provoke substantial inflammatory responses in this context [116]. Therefore, an interesting mechanism may involve oncogenes stimulating ROS generation, thereby creating a detrimental cycle that exacerbates cancer development.

### 5.3 Thyroid Stimulating Hormone (TSH)

There may be additional mechanisms that contribute to the production of ROS in thyroid cancer, thereby inducing a state of oxidative stress. Actually, the thyroid gland is an organ in which oxidative stress is continuously generated [123]. In this process, TSH binds to the TSH receptor (TSHR) on thyroid epithelial cells, consequently stimulating the production of H_2_O_2_ [124]. H_2_O_2_ is crucial for the biosynthesis of TH, acting as an electron acceptor at every stage of the process [125]. The primary implication of this finding is that increased production of H_2_O_2_, which is associated with a strengthened formation of free radicals, occurs concurrently with elevated serum TSH levels [126]. Therefore, TSH stimulation can lead to goitrogenesis and may contribute to the occurrence of thyroid cancer, at least in part, through the mechanism of oxidative stress [127]. This observation aligns with previous findings indicating that oxidative stress creates favorable conditions for the proliferation of thyroid cells [128].

## 6 Underlying Mechanisms of ROS in Thyroid Cancer

Existing research has demonstrated that ROS, especially H_2_O_2_, can activate the MAPK signaling pathway and subsequently promote cell proliferation and migration [129]. In addition, studies have also shown that the NF‐κB and PI3K/AKT pathways can be activated by ROS [130, 131]. These findings suggest a direct role for ROS in modulating key intracellular pathways that are implicated in human tumorigenesis.

The overactivation of the MAPK, PI3K/AKT, and NF‐κB signaling pathways constitutes a fundamental mechanism in thyroid tumorigenesis and progression [132]. It is thus plausible to hypothesize that ROS may contribute to thyroid cancer occurrence through these pathways. Given that genetic alterations in the MAPK and PI3K/AKT pathways are significant drivers of their activation [133, 134], it would be valuable to observe how ROS interacts with these genetic modifications to facilitate the initiation and progression of thyroid cancer. Analysis of data from TCGA has revealed a downregulation of 8‐oxoguanine DNA glycosylase (OGG1) levels in PTC exhibiting high MAPK1 expression, indicating that oxidative DNA damage plays a potential role in the pathogenesis of thyroid cancer.

Well‐differentiated PTC is primarily driven by mutually exclusive mutations that activate the MAPK pathway [113]. Among these mutations, BRAF^V600E^ mutations are the most prevalent, followed by RAS mutations and RET rearrangements [135]. Clinically, BRAF^V600E^ is correlated with an increased recurrence rate and decreased survival [136]. Previous studies have established the critical role of BRAF^V600E^ in the formation of tumors. However, the inhibition of the MAPK pathway is not consistently effective, particularly in the redifferentiation of radioiodine‐resistant tumors, suggesting the existence of additional mechanisms that contribute to BRAF^V600E^ adaptive resistance. In this context, transforming growth factor‐β (TGF‐β) has been shown to play a pivotal role in the process [121, 137].

TGF‐β is frequently overexpressed in thyroid cancer and serves as a potent pro‐oncogenic and pro‐metastatic factor [138]. Previous studies have shown that TGF‐β plays a leading role as a local modulator of the thyroid by inhibiting differentiation and growth in certain species [139]. The expression of BRAF^V600E^ induces TGF‐β1 generation, which initiates an autocrine loop driven by TGF‐β that partially mediates the role of the BRAF^V600E^ protein; it specifically regulates the promotion of cell migration, invasiveness, and the downregulation of sodium/iodide symporter (NIS) expression [140]. ROS can stimulate the generation of matrix metalloproteinases (MMPs), TGF‐β1, and other cytokines. Recent studies have demonstrated that abnormal production of MMPs and inflammatory factors is a main mechanism by which BRAF mutations promote the pathogenesis of thyroid cancer [119], with TGF‐β1 playing a pivotal role in this process [121, 140]. On the other hand, one study has shown that elevated levels of ROS could paradoxically induce the degradation of BRAF mutants under in vitro conditions [141]. Therefore, the interactions among ROS, TGF‐β1, and BRAF mutants may play a complex role in the pathogenesis of thyroid cancer.

## 7 Discussion

Obesity, recognized as a multifactorial metabolic disorder with complex pathophysiology, currently afflicts approximately 13% of the global population. Epidemiological projections suggest this prevalence may escalate to 20% among adults worldwide by 2030 should current trajectories persist [142], establishing obesity as a critical 21st‐century public health crisis [143]. Substantial evidence from systematic reviews identifies obesity as an established risk factor for oncogenesis, demonstrating significant associations with multiple malignancies including breast, esophageal, gallbladder, colorectal, and thyroid cancers [144]. The well‐established association between obesity and thyroid carcinogenesis (Table 1) has prompted intensive investigation into adipocytokine‐mediated mechanisms. Emerging evidence highlights the dysregulation of adipokine homeostasis—particularly reduced adiponectin and elevated leptin levels in obesity—as critical mediators of thyroid cancer pathogenesis through dual modulation of cellular proliferation, apoptosis, invasiveness, and angiogenesis. While current research has systematically characterized the molecular interplay between specific adipokines (e.g., leptin, adiponectin) and thyroid cancer progression through canonical signaling pathways (JAK/STAT, PI3K/AKT), the precise downstream signaling pathways and the relationship between obesity and metastatic spread remain poorly understood. Therefore, further studies are necessary to bridge the gap between in vivo and in vitro observations, thereby enhancing the physiological relevance of experimental results. Additional animal model studies, such as those utilizing genetically engineered murine models (e.g., adiponectin/leptin knockouts) coupled with longitudinal clinical cohorts, will be particularly valuable in this regard. Moreover, obesity is related to elevated levels of circulating pro‐inflammatory markers, such as TNF‐α and IL‐6. These adipocytokines demonstrate multifaceted oncogenic potential through their ability to stimulate angiogenesis [156] and induce genomic instability via DNA damage mechanisms [157], thereby initiating and promoting carcinogenic progression [158, 159]. The chronic inflammatory milieu created by these adipocytokines additionally exacerbates metabolic dysregulation, manifesting as insulin resistance and compensatory hyperinsulinemia—established risk factors for neoplastic development. Notably, the clinical implications of obesity extend beyond oncogenesis, showing a significant positive correlation with multiple chronic disorders such as hypertension, diabetes, and hyperlipidemia.

**TABLE 1:** Summary of the association between obesity and thyroid cancer risk.

Year	Author	Types of design	Study population	Main findings	References
2022	Shin A et al.	Cohort study	13 studies, 538,857 cases	While higher BMI is related to an elevated risk of thyroid cancer, underweight BMI may be associated differently with sex and histological subtypes.	[145]
2022	Jang Y et al.	Prospective cohort study	65,639 cases	Obesity indexes are related to an increased risk of thyroid cancer in women.	[146]
2021	Recalde M et al.	Prospective cohort study	3,658,417 cases	Higher BMI is positively associated with the risk of several cancers.	[147]
2021	O'Neill RJ et al.	Meta‐analysis	15 studies, 35,237 cases	Elevated BMI is significantly associated with numerous adverse presenting features of PTC.	[148]
2020	An SY et al.	Matched case–control	4977 TC cases and 19,908 controls	The incidence of overweight or obesity in the thyroid cancer group is higher than that in the control group.	[149]
2020	Zhao J et al.	Meta‐analysis	30 studies, 2174 TC cases and 1807 controls	Elevated levels of adipokines are closely related to thyroid cancer.	[78]
2020	Fussey JM et al.	Mendelian randomization	379,708 cases, contains 425 TC cases and 1812 controls	The results do not confirm a causal relationship between obesity and benign nodular thyroid disease or thyroid cancer.	[150]
2019	Kwon H et al.	Cohort study	255,051 cases	Excessive obesity is an independent risk factor for thyroid cancer.	[57]
2019	He Q et al.	Cohort study	10,668 TC cases and 11,858 controls	BMI, BSA and BF% are positively associated with the risk of DTC, which may be influenced by age and gender.	[151]
2018	Sadeghi H et al.	Meta‐analysis	17 studies	Obesity, overweight, and radiation exposure are significantly associated with an increased risk of thyroid cancer.	[152]
2018	Hidayat K et al.	Meta‐analysis	56 studies, 27,559 cancer cases	Higher body fatness at a young age increases the risk of various cancers later in life.	[153]
2016	Zagzag J et al.	Cohort study	473 cases	BMI does not play a role in the initial detection of WDTC patients.	[154]
2015	Schmid D et al.	Meta‐analysis	21 studies, 12,199 TC cases	Obesity was positively correlated with papillary, follicular, and anaplastic thyroid carcinoma, and negatively correlated with medullary thyroid carcinoma.	[56]
2014	Kitahara CM et al.	Cohort study	321,085 cases	Taller height and greater BMI in childhood are correlated with an increased risk of thyroid carcinoma.	[155]

Abbreviations: BF%, body fat percentage; BMI, body mass index; BSA, body surface area; PTC, papillary thyroid cancer; TC, thyroid cancer; WDTC, well‐differentiated thyroid cancer.

Although the majority of hypotheses regarding the role of oxidative stress in thyroid tumors, as discussed above, remain untested, there is compelling evidence suggesting that increased oxidative stress serves as a significant risk factor closely associated with thyroid tumorigenesis. ROS may play a complex and paradoxical role in cancer progression; specifically, low levels of ROS can promote the proliferation or invasion of cancer, whereas high levels of ROS can induce cell apoptosis, thereby inhibiting tumor progression [160, 161]. DUOX1 and NOX4 are involved in the generation of ROS in the context of thyroid tumor development, which opens the way for further investigation into their role in the mechanisms that lead to genetic aberrations (Figure 3). Dysregulation of the expression of these enzymes poses a threat to genomic stability, consequently affecting cell survival. Notably, DUOX1 has been shown to promote persistent DNA damage in human thyrocytes following irradiation, which is recognized as a significant risk factor for thyroid cancer [162]. Similarly, NOX4 appears to play a crucial role in promoting thyroid cancer development, especially in PTC cases harboring the BRAF^V600E^ mutation, which is associated with refractoriness to radioactive iodine treatment and cellular dedifferentiation [115]. Recent studies have suggested that NOX4 may serve as a potential therapeutic target due to its involvement in the repression of NIS. Cancer cells are characterized by elevated ROS levels and enhanced aerobic glycolysis, which can be counteracted by increased antioxidant defenses [163]. Therefore, the modulation of intracellular ROS generation may represent a promising new approach in cancer therapy. A more detailed discussion of existing treatment strategies that have demonstrated efficacy in research is beyond the scope of this article, but potential approaches can generally be divided into three main areas: (a) reversing and/or preventing obesity, (b) targeting obesity‐associated metabolic disease, and (c) targeting adipose tissue inflammation. Physical exercise, weight reduction, bariatric surgery, and the adoption of a balanced diet have shown tremendous promise in disrupting the underlying link between obesity and cancer [164, 165, 166]. One of the most obvious features during weight loss via either dieting or surgery is the decreased number of infiltrating macrophages in both adipose tissue and obesity‐related cancer tissues. Moreover, certain commonly prescribed medications, including metformin, statins, and nonsteroidal anti‐inflammatory drugs, may contribute to the mitigation of obesity‐induced inflammation and metabolic dysfunction. However, the extent to which these medications confer protective effects against the development of thyroid cancer remains uncertain. As with most targeted therapies, it is essential to identify specific individuals at high risk in order to optimize the potential therapeutic benefits of these pharmacological interventions [167, 168, 169].

Emerging evidence suggests that the obesity‐thyroid cancer nexus involves a complex interplay of multiple pathophysiological mechanisms rather than a singular pathway. Given this mechanistic plurality, contemporary therapeutic paradigms emphasize multimodal strategies concurrently targeting inflammatory mediators, metabolic derangements, and hormone signaling pathways. Sustained research endeavors elucidating the pathophysiological mechanisms through which obesity drives tumorigenesis, promotes malignant progression, and enhances metastatic dissemination may pave the way for innovative therapeutic interventions targeting adiposity‐associated oncogenic pathways.

## Author Contributions

**Bo‐Tao Zhang:** conceptualization, writing – original draft, writing – review and editing, funding acquisition, software. **Mao Guo:** writing – original draft, software, resources. **Liu‐Rui Yang:** software, resources. **Yang Zeng:** conceptualization, project administration, writing – review and editing. **Jun Jiang:** conceptualization, project administration, writing – review and editing, funding acquisition.

## Conflicts of Interest

The authors declare no conflicts of interest.

## Data Availability

Data sharing is not applicable to this article as no new data were created or analyzed in this study.
